# Supplementary material for: The inhibition mechanism of the SUR2A-containing KATP channel by a regulatory helix
Source: Nat Commun. 2023 Jun 17;14:3608. doi: 10.1038/s41467-023-39379-4 (PMC10276813; doi:10.1038/s41467-023-39379-4)
Supplement: Supplementary file 1 — Supplementary Information [file 41467_2023_39379_MOESM1_ESM.pdf]

Supplementary information for

The inhibition mechanism of the SUR2A-containing K_{ATP} channel by a regulatory helix

Dian Ding, Tianyi Hou, Miao Wei, Jing-Xiang Wu, Lei Chen*

*To whom correspondence should be addressed: Lei Chen (chenlei2016@pku.edu.cn)

This file includes:
Supplementary Figs. 1-11
Supplementary Table 1

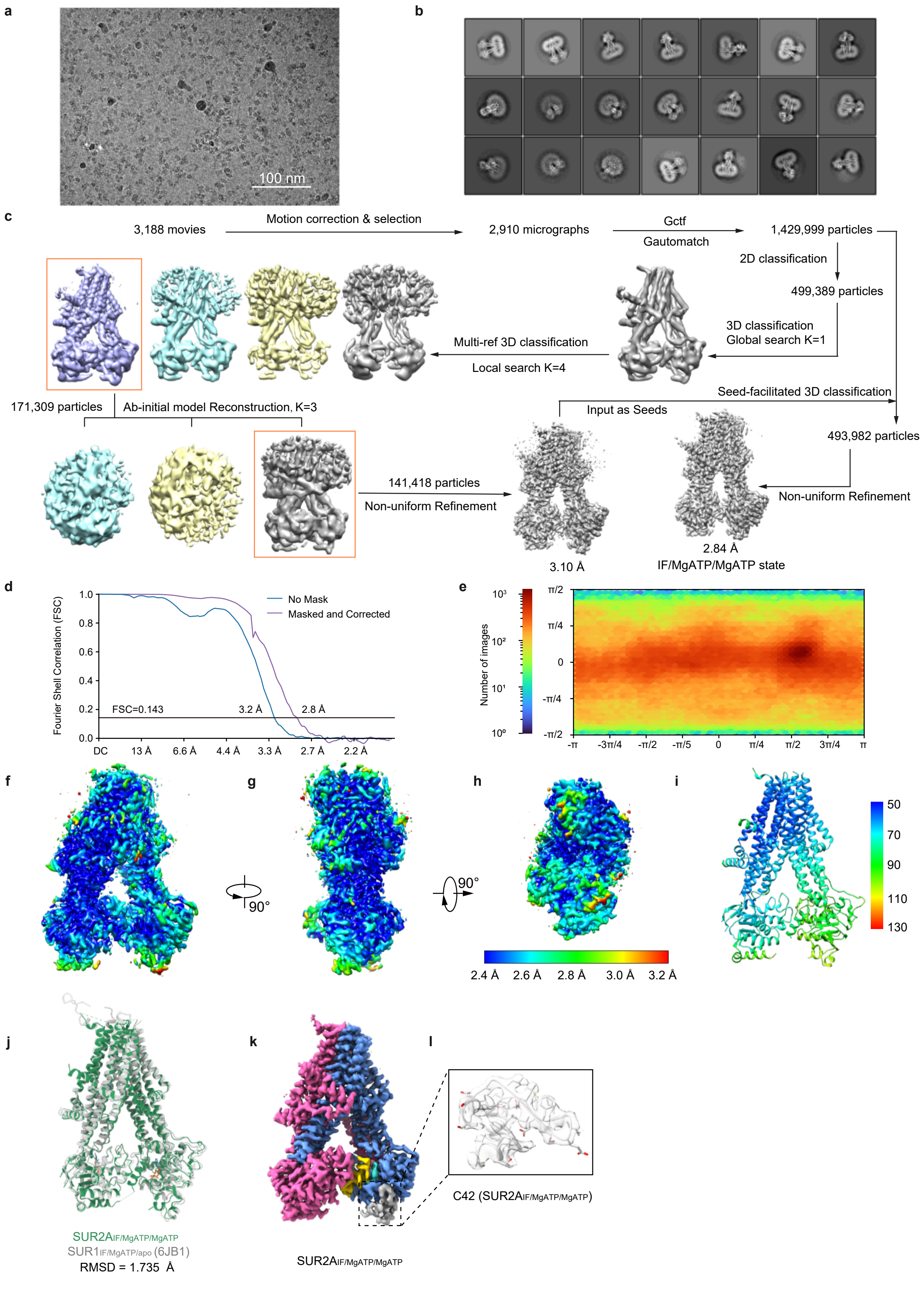

Supplementary Fig. 1: Workflow for cryo-EM data processing of SUR2A in complex with Mg-ATP and RPG. a Representative image from a dataset consisting of 2,910 motion corrected micrographs. **b** Two-dimensional class averages of the sample. **c** EM data processing workflow. **d** Resolution estimation of the SUR2A map, based on the criterion of the FSC 0.143 cut-off. **e** Angular distribution of the final reconstruction. **f–h** Local resolution maps in different views. **i** $SUR2A_{IF/MgATP/MgATP}$ model colored by B-factor. **j** Structural comparison between $SUR2A_{IF/MgATP/MgATP}$ state and $SUR1_{IF/MgATP/apo}$ state (amino acids 223 to 1579). **k** Cryo-EM map of $SUR2A_{IF/MgATP/MgATP}$. The EM density of C42 is colored in white and the color scheme of the remaining parts is the same as that in Figure 1. **l** Close-up view of the cryo-EM density of C42 of $SUR2A_{IF/MgATP/MgATP}$ boxed as dashed in (**k**).

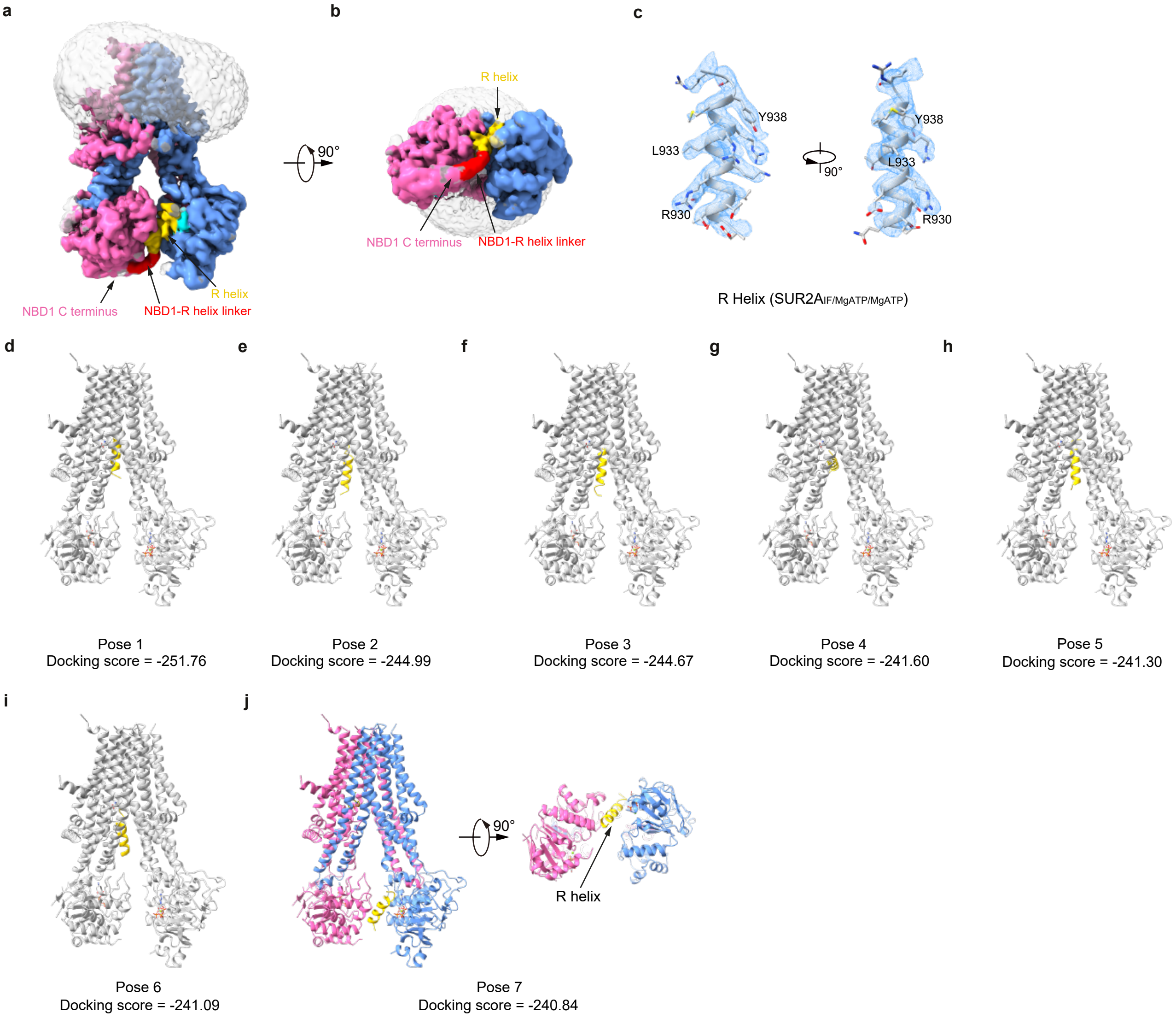

Supplementary Fig. 2: The modeling of R helix in SUR2A$_{IF/MgATP/MgATP}$. **a** Unsharpened full map of SUR2A$_{IF/MgATP/MgATP}$ contoured at 0.30 level (5.0 σ). The continuous NBD1-R helix linker is colored in red. **b** The bottom view of SUR2A$_{IF/MgATP/MgATP}$ contoured at 0.30 level (5.0 σ). **c** Cryo-EM density of R helix in the SUR2A$_{IF/MgATP/MgATP}$ state. The map was contoured at 0.62 level (4.5 σ). **d-i** Top 6 docking poses of the R helix (yellow) onto SUR2A (gray) calculated with HDOCK. The binding poses of R helix in these dockings are impossible because R helix could not physically connect to NBD1 or TMD2 due to sterical clashes or spatial restraint of linkers. **j** The 7th docked model of the R helix, colored the same as in Figure 1. This docking model is the same as that experimentally determined in SUR2A$_{IF/MgATP/MgATP}$ in this work.

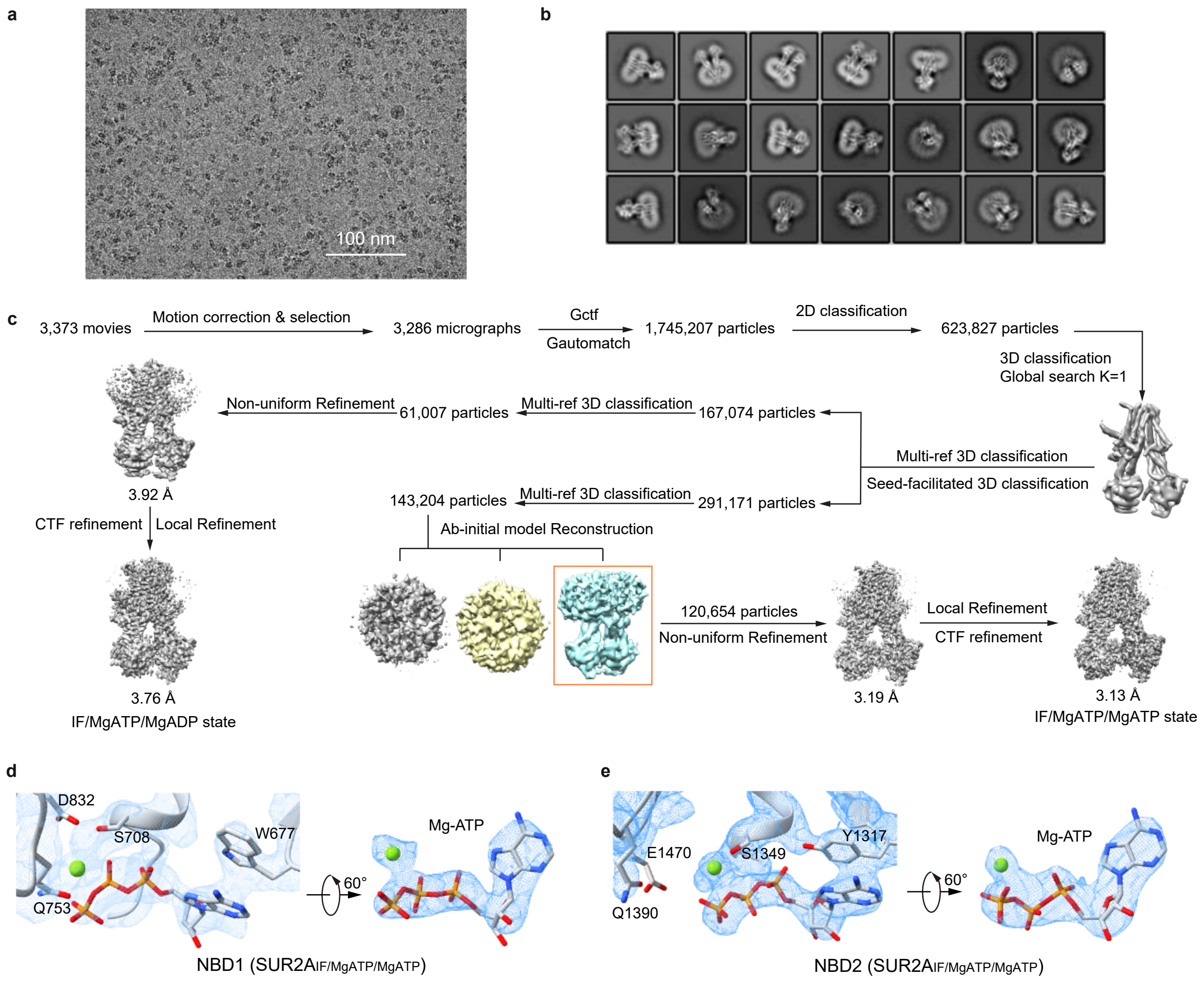

Supplemetary Fig. 3: Workflow for cryo-EM data processing of SUR2A in complex with Mg-ATP, Mg-ADP, and RPG. a Representative image from a dataset consisting of 3,286 motion corrected micrographs. **b** Two-dimensional class averages of the SUR2A. **c** EM data processing workflow. **d-e** Close-up views of EM densities at NBD1 (**d**) and NBD2 (**e**) of SUR2A$_{IF/MgATP/MgATP}$ state. The maps were contoured at 0.87 level (9.3 σ)(**d**) and 0.70 level (7.5 σ)(**e**), respectively.

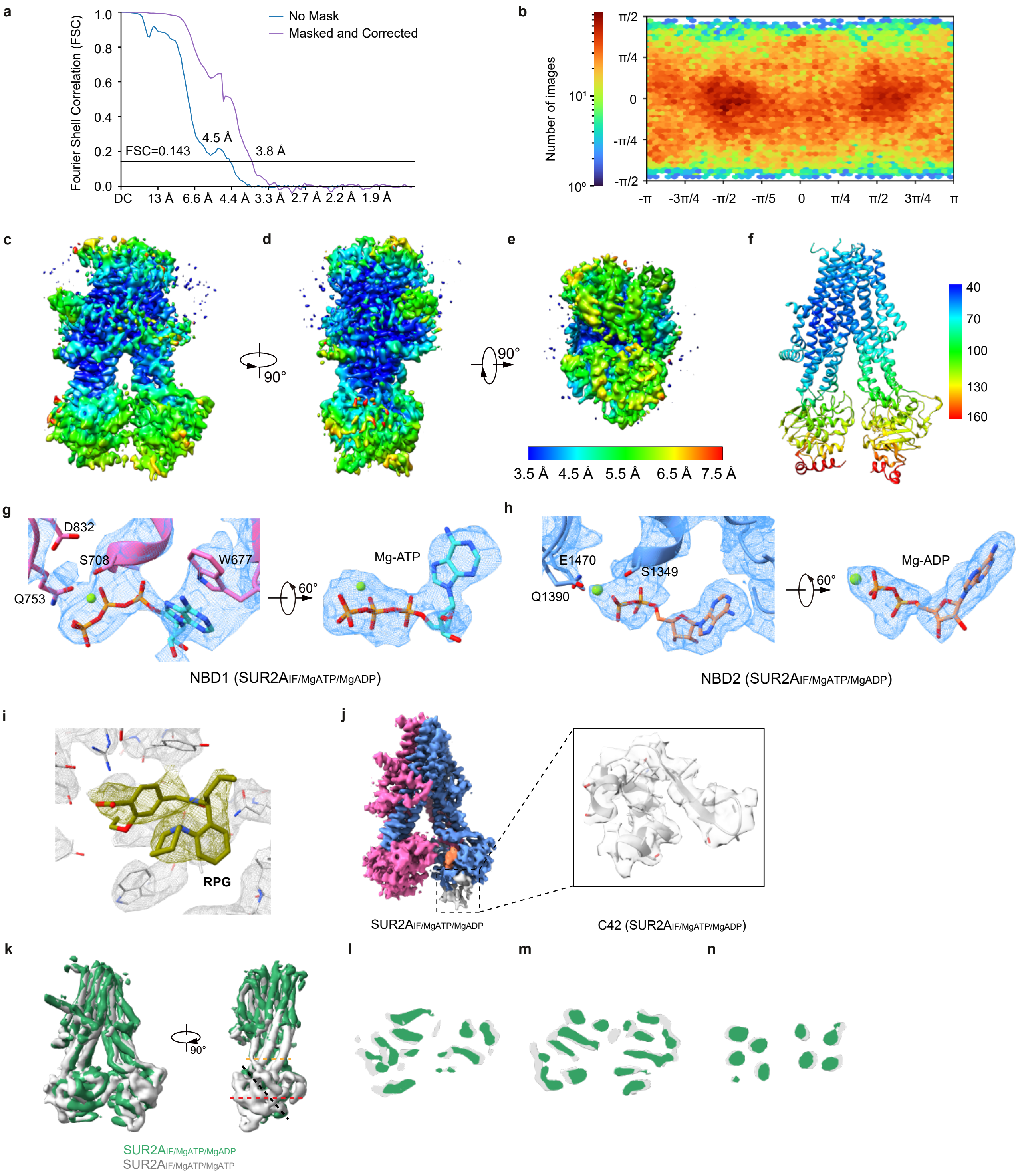

Supplementary Fig. 4: Structure analysis of SUR2AIF/MgATP/MgADP. a Resolution estimation of the SUR2AIF/MgATP/MgADP, based on the criterion of the FSC 0.143 cut-off. **b** Angular distribution of the final reconstruction. **c-e** Local resolution map of the final density map. **f** SUR2AIF/MgATP/MgADP model colored by B-factor. **g-h** Close-up views of EM densities at NBD1 (**g**) and NBD2 (**h**) of SUR2AIF/MgATP/MgADP state. The maps were contoured at 0.60 level (8.8 σ)(**d**) and 0.48 level (7.1 σ)(**e**), respectively. **i** RPG density in the IS-binding site of SUR2AIF/MgATP/MgADP. The map is shown as mesh. The protein and RPG are shown as sticks. The map was contoured at 0.69 level (10.1 σ). **j** Cryo-EM density of C42 of SUR2AIF/MgATP/MgADP state. **k** The alignment of cryo-EM maps of SUR2AIF/MgATP/MgATP state (gray) and SUR2AIF/MgATP/MgADP state (green). Both maps were low-pass filtered at 6 Å. SUR2AIF/MgATP/MgATP state and SUR2AIF/MgATP/MgADP state were contoured at 0.40 level (3.9 σ) and 0.37 level (3.9σ), respectively. **l-n** The cut-open view at the position of the cross-section indicated by the black dashes (**l**), red dashes (**m**) and yellow dashes (**n**) in (**k**).

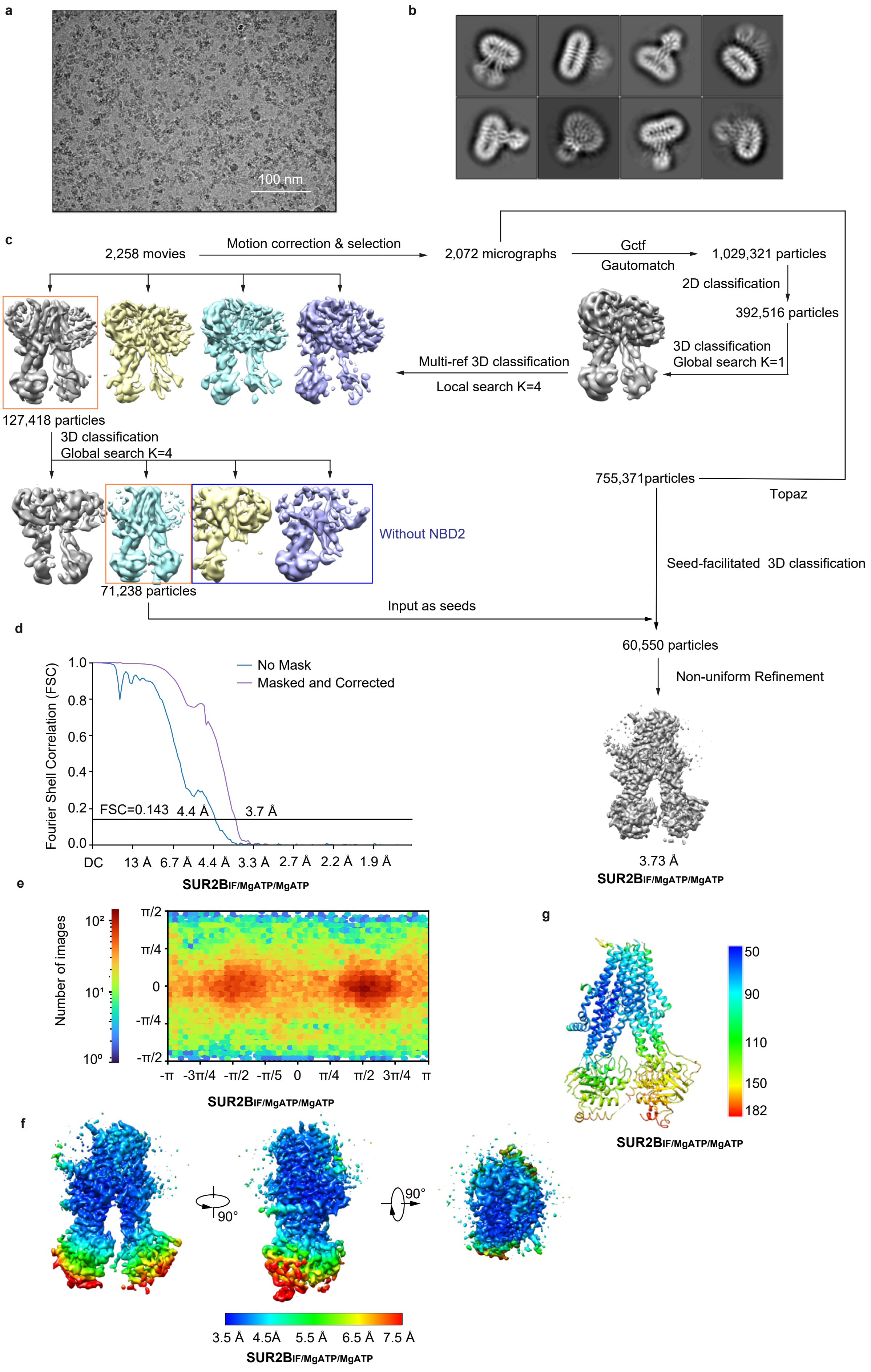

Supplementary Fig. 5: Workflow for cryo-EM data processing of SUR2B in complex with Mg-ATP and RPG. **a** Representative image from a dataset consisting of 2,072 motion corrected micrographs. **b** Two-dimensional class averages of SUR2B in complex with Mg-ATP and RPG. **c** EM data processing workflow. 3D classes without NBD2 densities were boxed in blue. **d** Resolution estimation of the $SUR2B_{IF/MgATP/MgATP}$ map, based on the criterion of the FSC 0.143 cut-off. **e** Angular distribution of the final reconstruction. **f** Local resolution map of the final density map. **g** The model of $SUR2B_{IF/MgATP/MgATP}$ colored by B factors.

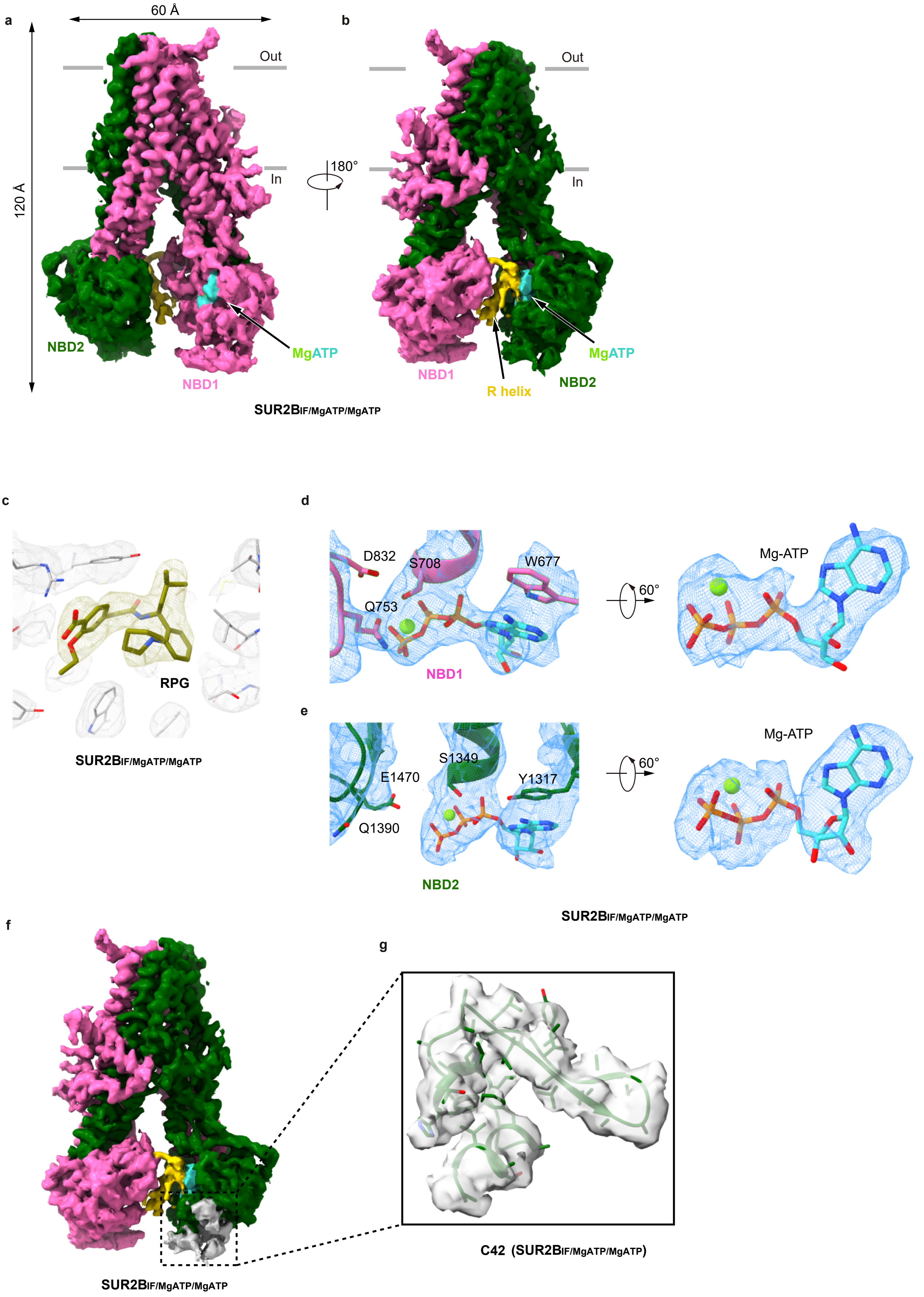

Supplementary Fig. 6: Cryo-EM maps of SUR2B in complex with RPG and MgATP. a-b The cryo-EM density map of SUR2B$_{IF/MgATP/MgATP}$, viewed from the side. The approximate position of the lipid bilayer is indicated by gray bars. TMD1-NBD1, TMD2-NBD2, R helix, RPG, Mg^{2+}, and ATP are colored in pink, dark green, yellow, olive, green, and cyan, respectively. **c** RPG density in the insulin secretagogue-binding site of SUR2B$_{IF/MgATP/MgATP}$. The map is shown as mesh. Protein and RPG are shown as sticks. The map was contoured at 0.26 level (4.1 σ). **d-e** Close-up views of EM densities at the degenerate site of NBD1 (**d**) and the consensus site of NBD2 (**e**). The map was contoured at 0.26 level (4.1 σ). **f-g** C42 of SUR2B$_{IF/MgATP/MgATP}$ is colored in silver (**f**) and the close-up view of C42 is shown in surface (**g**). The map was contoured at 0.26 level (4.1 σ).

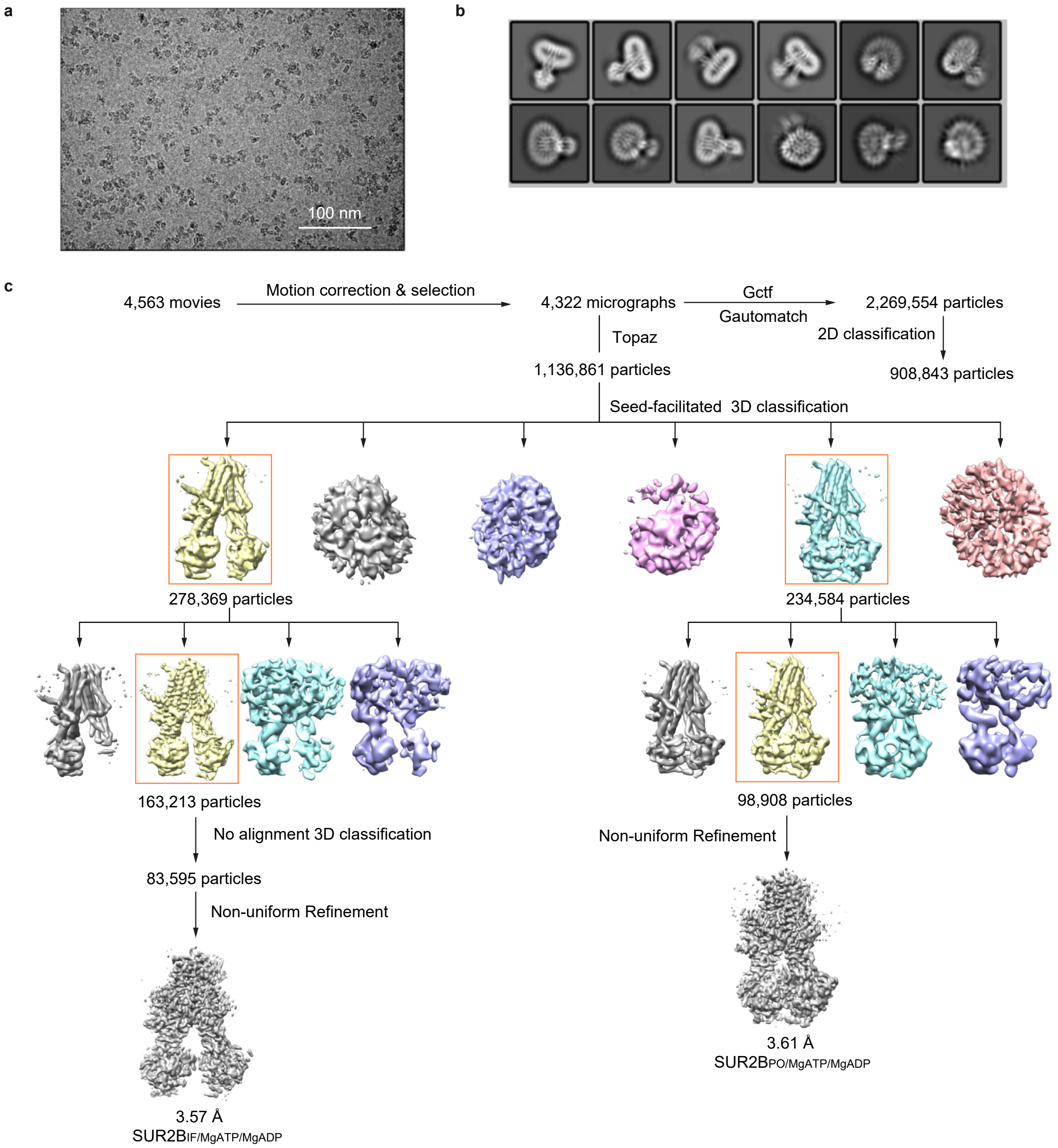

Supplementary Fig. 7: Workflow for cryo-EM data processing of SUR2B in the presence of RPG, MgATP, and MgADP. **a** Representative image from a dataset consisting of 4,322 motion corrected micrographs. **b** Two-dimensional class averages of SUR2B in complex with Mg-ATP, Mg-ADP, and RPG. **c** EM data processing workflow.

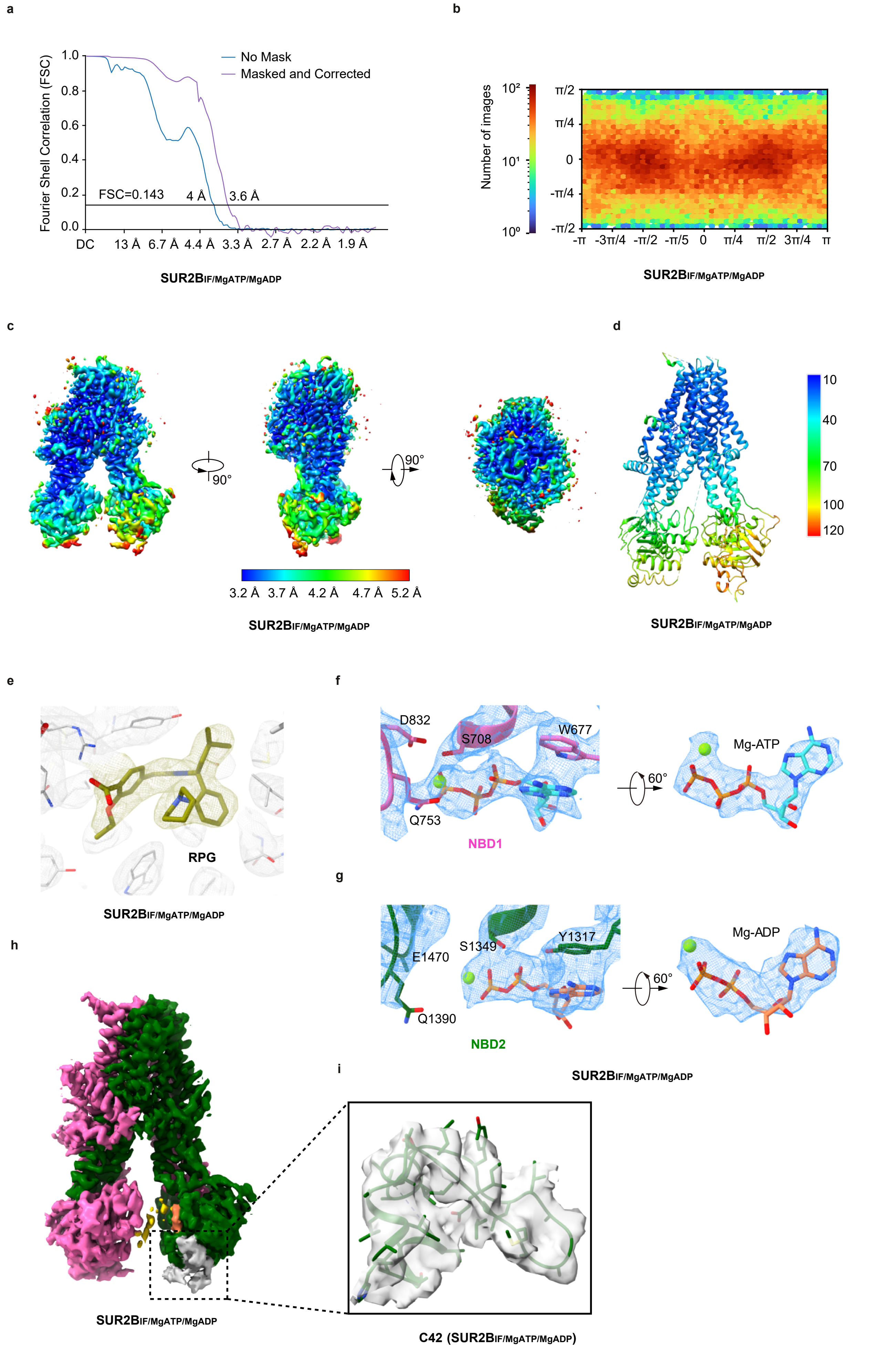

Supplementary Fig. 8: Structure analysis of SUR2BIF/MgATP/MgADP. a Resolution estimation of the SUR2BIF/MgATP/MgADP map, based on the criterion of the FSC 0.143 cut-off. **b** Angular distribution of the final reconstruction. **c** Local resolution estimation of the final density map. **d** The model of SUR2BIF/MgATP/MgADP colored by B factors. **e** RPG density in the insulin secretagogue-binding site of SUR2BIF/MgATP/MgADP. The map is shown as mesh. Protein and RPG are shown as sticks.The map was contoured at 0.32 level (3.7 σ). **f-g** Close-up views of EM densities at the degenerate site of NBD1 (**f**) and the consensus site of NBD2 (**g**).The map was contoured at 0.32 level (3.7 σ). **h-i** C42 of SUR2BIF/MgATP/MgADP is colored in silver (**h**) and the close-up view of C42 is shown in surface (**i**).The map was contoured at 0.32 level (3.7 σ).

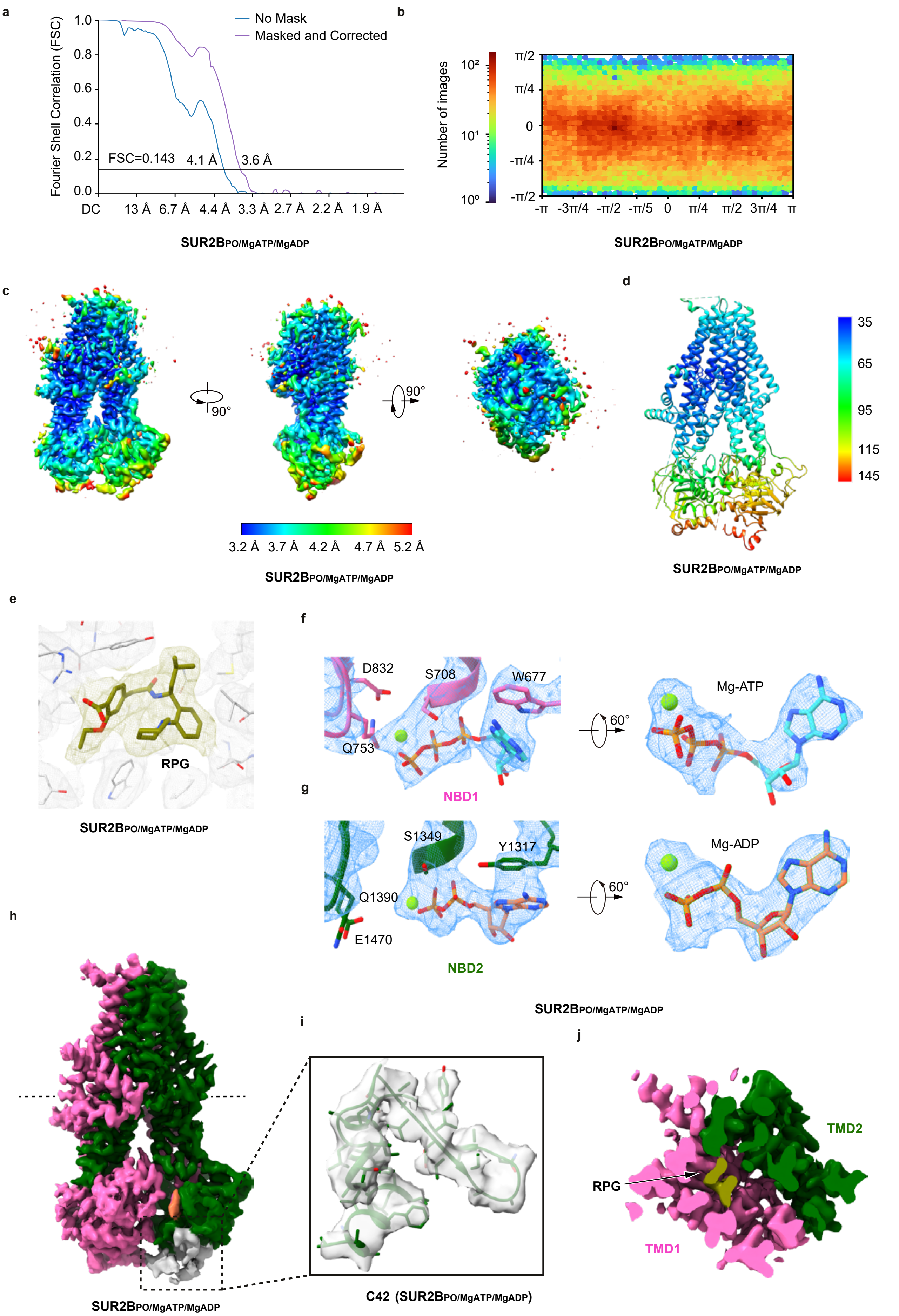

Supplementary Fig. 9: Structure analysis of SUR2BPO/MgATP/MgADP. **a** Resolution estimation of the SUR2BPO/MgATP/MgADP map, based on the criterion of the FSC 0.143 cut-off. **b** Angular distribution of the final reconstruction. **c** Local resolution map of the final density map **d** Model of SUR2BPO/MgATP/MgADP colored by B factors. **e** RPG density in the insulin secretagogue-binding site of SUR2BPO/MgATP/MgADP. The map is shown as mesh. Protein and RPG are shown as sticks.The map was contoured at 0.31 level (3.9 σ) . **f-g** Close-up views of EM densities at the degenerate site of NBD1 (**f**) and the consensus site of NBD2 (**g**).The map was contoured at 0.31 level (3.9 σ). **h-i** C42 of SUR2BPO/MgATP/MgADP is colored in silver (**h**) and the close-up view of C42 is shown in surface (**i**).The map was contoured at 0.31 level (3.9 σ). **j** The cut-open view of the transmembrane domain. The approximate position of the section is indicated as the dashed line in (**h**). RPG, TMD1, and TMD2 are colored in olive, pink, and dark green.

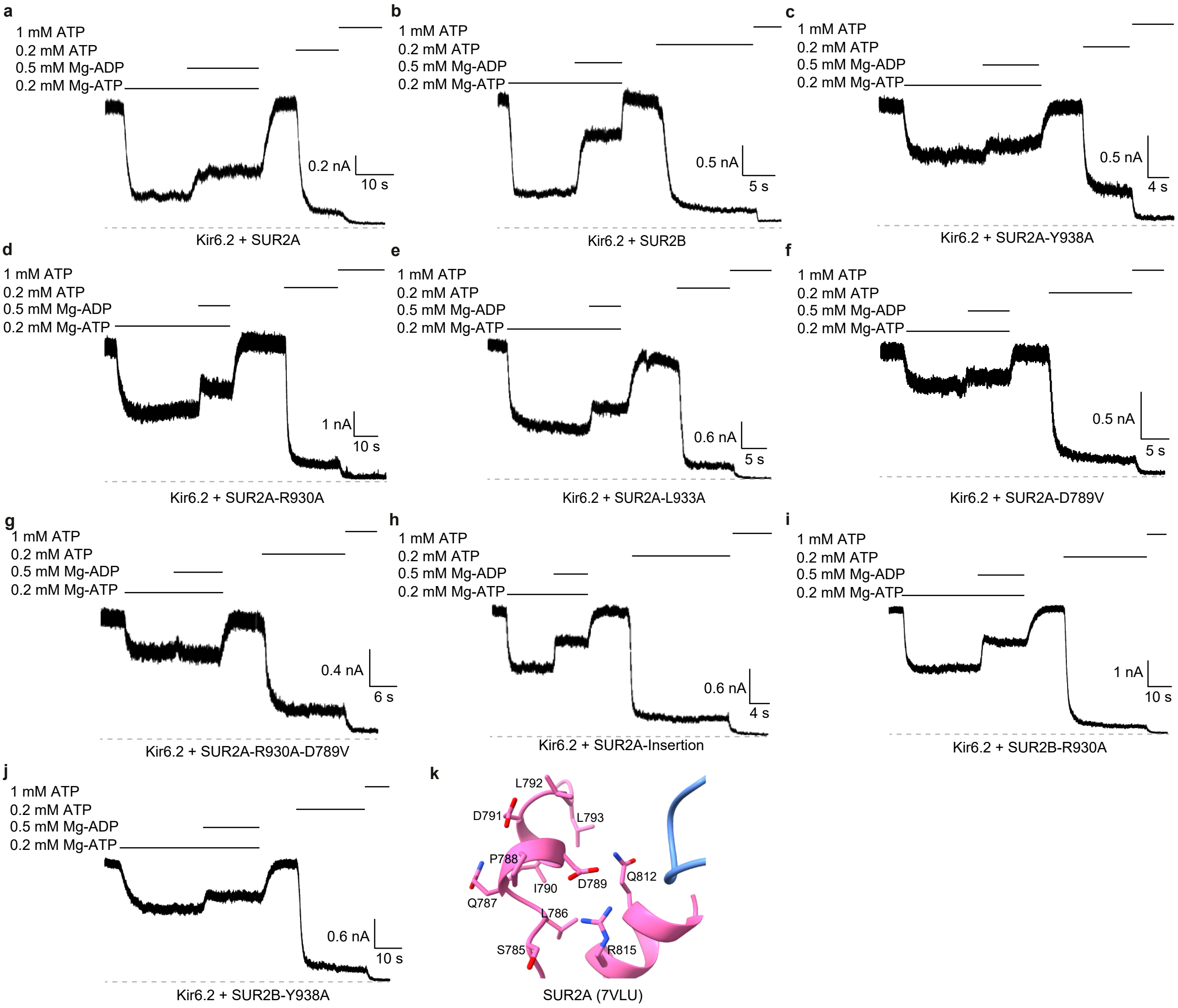

Spplementary Fig. 10: Inside-out currents of various K_{ATP} constructs. a-j Inside out currents of wt SUR2A construct (**a**), wt SUR2B construct (**b**), SUR2A-Y938A construct (**c**), SUR2A-R930A construct (**d**), SUR2A-L933A construct (**e**), SUR2A-D789V construct (**f**), SUR2A-R930A-D789V construct (**g**), SUR2A-Insertion construct (**h**), SUR2B-R930A construct (**i**) and SUR2B-Y938A construct (**j**). The dotted lines indicate the zero current positions. **k** Close-up view of D789 of $SUR2A_{OD/ATP/ADP}$. Residues within 4 Å of D789 are shown as sticks. NBD1 and NBD2 are colored in pink and blue, respectively.

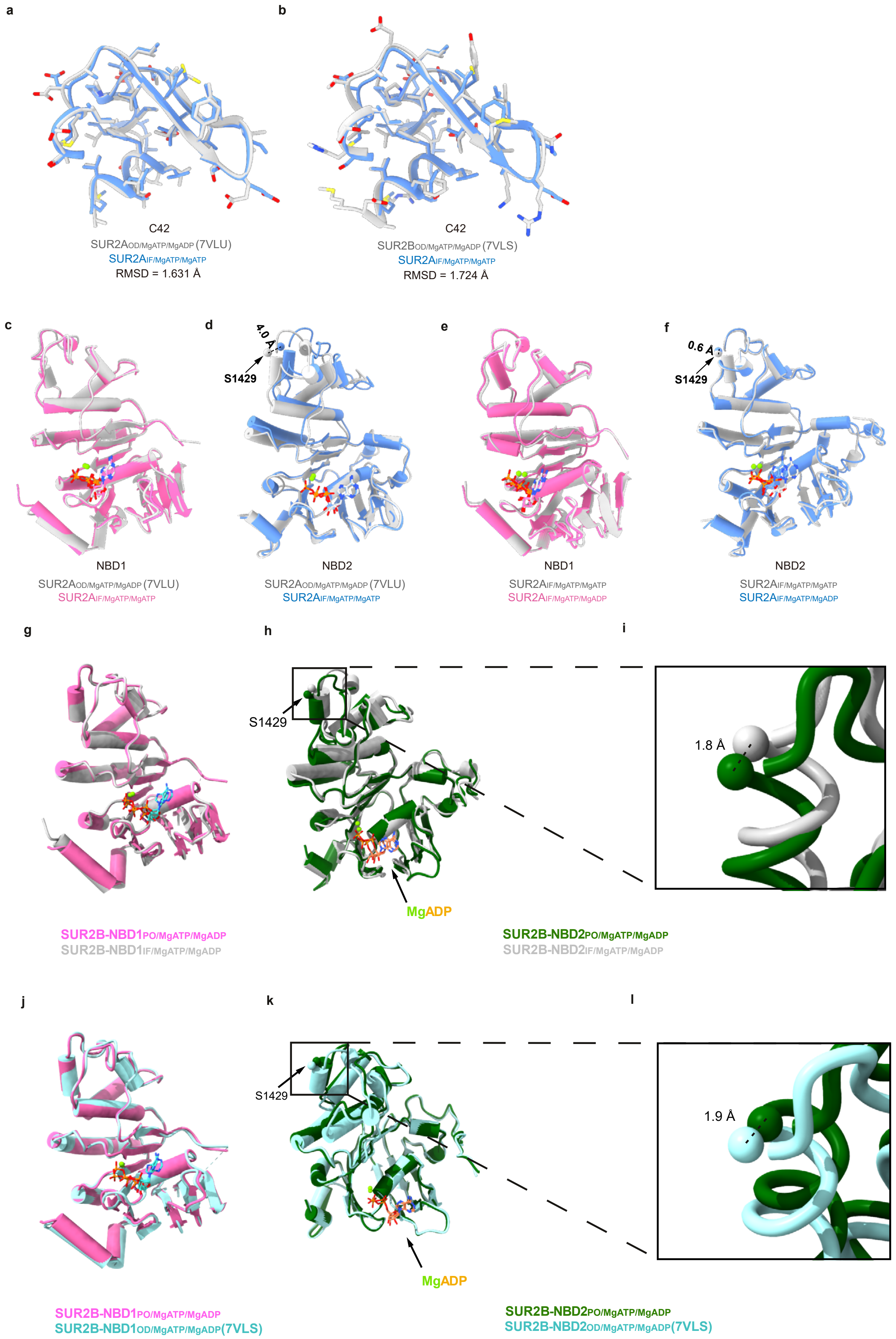
a
b
C42
SUR2AOD/MgATP/MgADP (7VLU)
SUR2AIF/MgATP/MgATP
RMSD = 1.631 Å
C42
SUR2BOD/MgATP/MgADP (7VLS)
SUR2AIF/MgATP/MgATP
RMSD = 1.724 Å
c
d
e
f
4.0 Å
S1429
0.6 Å
S1429
NBD1
SUR2AOD/MgATP/MgADP (7VLU)
SUR2AIF/MgATP/MgATP
NBD2
SUR2AOD/MgATP/MgADP (7VLU)
SUR2AIF/MgATP/MgATP
NBD1
SUR2AIF/MgATP/MgATP
SUR2AIF/MgATP/MgADP
NBD2
SUR2AIF/MgATP/MgATP
SUR2AIF/MgATP/MgADP
g
h
i
S1429
1.8 Å
MgADP
SUR2B-NBD1PO/MgATP/MgADP
SUR2B-NBD1IF/MgATP/MgADP
SUR2B-NBD2PO/MgATP/MgADP
SUR2B-NBD2IF/MgATP/MgADP
j
k
l
S1429
1.9 Å
MgADP
SUR2B-NBD1PO/MgATP/MgADP
SUR2B-NBD1OD/MgATP/MgADP(7VLS)
SUR2B-NBD2PO/MgATP/MgADP
SUR2B-NBD2OD/MgATP/MgADP(7VLS)

Supplementary Fig. 11: Structural comparisons between different states of SUR2A and SUR2B. a Structural comparisons between different states of SUR2A and SUR2B. (**a**) Structural comparison of the C-terminal 42 residues between SUR2A$_{\text{OD/MgATP/MgADP}}$ (gray, PDB ID: 7VLU) and SUR2A$_{\text{IF/MgATP/MgATP}}$ (blue). (**b**) Structural comparison of the C-terminal 42 residues between SUR2B$_{\text{OD/MgATP/MgADP}}$ (gray, PDB ID: 7VLS) and SUR2A$_{\text{IF/MgATP/MgATP}}$ (blue). (**c**) Structural comparison of NBD1 between SUR2A$_{\text{OD/MgATP/MgADP}}$ (gray, PDB ID: 7VLU) and SUR2A$_{\text{IF/MgATP/MgATP}}$ (pink). NBD structures are aligned according to the β sheet-rich subdomains. (**d**) Structural comparison of NBD2 between SUR2A$_{\text{OD/MgATP/MgADP}}$ (gray, PDB ID: 7VLU) and SUR2A$_{\text{IF/MgATP/MgATP}}$ (blue). Cα distance of S1429 between the two states is shown as a dashed line. NBD structures are aligned according to the β sheet-rich subdomains. (**e**) Structural comparison of NBD1 between SUR2A$_{\text{IF/MgATP/MgADP}}$ (pink) and SUR2A$_{\text{IF/MgATP/MgATP}}$ (gray). NBD structures are aligned according to the β sheet-rich subdomains. (**f**) Structural comparison of NBD2 between SUR2A$_{\text{IF/MgATP/MgADP}}$ (blue) and SUR2A$_{\text{IF/MgATP/MgATP}}$ (gray). Cα distance of S1429 between the two states is shown as a dashed line. NBD structures are aligned according to the β sheet-rich subdomains. (**g**) Structural comparison of NBD1 between SUR2B$_{\text{IF/MgATP/MgADP}}$ (gray) and SUR2B$_{\text{PO/MgATP/MgADP}}$ (pink). (**h**) Structural comparison of NBD2 between SUR2B$_{\text{IF/MgATP/MgADP}}$ (gray) and SUR2B$_{\text{PO/MgATP/MgADP}}$ (green). (**i**) Close-up view of conformational changes of SUR2B NBD2. Displacement of the S1429 Cα atom is denoted. NBD structures are aligned according to the β sheet-rich subdomains. (**j**) Structural comparison of NBD1 between SUR2B$_{\text{OD/MgATP/MgADP}}$ (cyan) and SUR2B$_{\text{PO/MgATP/MgADP}}$ (pink). (**k**) Structural comparison of NBD2 between SUR2B$_{\text{OD/MgATP/MgADP}}$ (cyan) and SUR2B$_{\text{PO/MgATP/MgADP}}$ (green). (**l**) The close-up view of conformational changes of SUR2B NBD2. Displacement of the S1429 Cα atom is denoted. NBD structures are aligned according to the β sheet-rich subdomains.

Supplementary Table 1
Cryo-EM data collection, refinement and validation statistics

	SUR2A IF/MgATP/MgATP	SUR2A IF/MgATP/MgADP
PDB ID	7Y1J	7Y1K
EMDB ID	EMD-33563	EMD-33564
**Data collection and processing**		
Magnification	105,000 ×	105,000 ×
Voltage (kV)	300	300
Electron exposure ($e^-/Å^2$)	52	52
Defocus range (μm)	-1.8 to -2.0	-1.8 to -2.0
Pixel size (Å)	0.834	0.834
Symmetry imposed	*C1*	*C1*
Initial particle images (no.)	1,429,999	1,745,207
Final particle images (no.)	493,982	61,007
Map resolution (Å)	2.8	3.8
FSC threshold	0.143	0.143
Map resolution range (Å)	250-2.8	250-3.8
**Refinement**		
Initial model used (PDB code)	6JB1	6JB1
Model resolution (Å)	2.8	3.7
FSC threshold	0.143	0.143
Model resolution range (Å)	250-2.8	250-3.7
Map sharpening B factor ($Å^2$)	-135.1	-140.6
Model composition		
Non-hydrogen atoms	9,487	8,510
Protein residues	1,204	1,164
Ligands	5	5
B factors ($Å^2$)		
Protein	48.62	88.82
Ligand	41.57	100.56
R.m.s. deviations		
Bond lengths (Å)	0.004	0.004
Bond angles (°)	0.490	0.548
Validation		
MolProbity score	1.32	1.74
Clashscore	5.82	7.2
Poor rotamers (%)	0.69	2.83
Ramachandran plot		
Favored (%)	99.16	98.17
Allowed (%)	0.84	1.83
Disallowed (%)	0.00	0.00
Model content	216-274	217-274
	282-324	282-327
	342-613	343-611
	665-728	666-728
	745-940	745-913
	966-1018	975-1018
	1025-1542	1028-1542

PDB ID<br>EMDB ID	SUR2B<br>IF/MgATP/MgATP<br>7Y1L<br>EMD-33565	SUR2B<br>IF/MgATP/MgADP<br>7Y1M<br>EMD-33566	SUR2B<br>PO/MgATP/MgADP<br>7Y1N<br>EMD-33567
**Data collection and processing**			
Magnification	105,000 ×	105,000 ×	105,000 ×
Voltage (kV)	300	300	300
Electron exposure (e^{-}/Å^2)	52	52	52
Defocus range (μm)	-1.8 to -2.0	-1.8 to -2.0	-1.8 to -2.0
Pixel size (Å)	0.834	0.834	0.834
Symmetry imposed	*C1*	*C1*	*C1*
Initial particle images (no.)	755,371	1,136,861	1,136,861
Final particle images (no.)	60,550	83,595	98,908
Map resolution (Å)	3.73	3.57	3.61
FSC threshold	0.143	0.143	0.143
Map resolution range (Å)	250-3.73	250-3.57	250-3.61
**Refinement**			
Initial model used (PDB code)	6JB1	6JB1	7VLR
Model resolution (Å)	3.6	3.5	3.5
FSC threshold	0.143	0.143	0.143
Model resolution range (Å)	250-3.6	250-3.5	250-3.5
Map sharpening B factor (Å^2)	-165.2	-149.0	-163.1
Model composition			
Non-hydrogen atoms	8,955	8,957	8,763
Protein residues	1,189	1,166	1,171
Ligands	5	5	5
B factors (Å^2)			
Protein	108.88	60.07	77.44
Ligand	116.55	71.79	86.56
R.m.s. deviations			
Bond lengths (Å)	0.005	0.003	0.005
Bond angles (°)	0.956	0.502	0.678
Validation			
MolProbity score	1.45	1.45	1.56
Clashscore	8.01	7.76	7.98
Poor rotamers (%)	1.03	0.33	1.30
Ramachandran plot			
Favored (%)	98.21	97.91	97.84
Allowed (%)	1.79	2.09	2.16
Disallowed (%)	0.00	0.00	0.00
Model content	216-274<br>282-324<br>342-611<br>666-728<br>745-913<br>924-940<br>968-1018<br>1027-1543	216-274<br>282-324<br>342-611<br>666-728<br>746-913<br>968-1018<br>1027-1531<br>1535-1541	217-275<br>283-327<br>341-610<br>665-728<br>744-912<br>969-1018<br>1028-1236<br>1241-1545
